# Vehicle avoidance: The hierarchy of visual attention towards animals, plants, and vehicles

**DOI:** 10.1371/journal.pone.0330475

**Published:** 2025-09-22

**Authors:** Chihiro Kioka, Kunihito Tobita

**Affiliations:** 1 Department of Sustainable System Sciences, Osaka Prefecture University, Sakai, Japan; 2 Department of Psychology, Osaka Metropolitan University, Sakai, Japan; Education University of Hong Kong, HONG KONG

## Abstract

The biophilia hypothesis posits that humans have an innate affinity for nature, with natural landscapes effortlessly capturing their attention, and a tendency to seek nature. The animate monitoring hypothesis suggests that humans have evolved to quickly detect and respond to animals for survival. The plant awareness disparity hypothesis argues that people notice plants less than animals due to perceptual biases and preferences. Based on these hypotheses, it was predicted that people’s visual attention would be superior towards animals, plants, and manufactured objects, in that order. This study investigated the hierarchy of visual attention towards animals (birds, mammals and humans), plants (fruit), and manufactured objects (vehicles) using a dot-probe task framework. The findings revealed no significant differences in reaction time or attentional bias for animal or plant stimuli. In contrast, perceptual processing was inhibited when viewing a vehicle and attentional avoidance occurred, resulting in slower reactions than to animals or plants. These findings offer partial support for the proposed hierarchy of visual attention, suggesting that while natural stimuli such as animals and plants receive comparable attention, some manufactured objects may elicit perceptual avoidance.

## Introduction

Frequent visits to green spaces are associated with a lower risk of attention deficit hyperactivity disorder [1], Alzheimer’s disease [2], depression, and hypertension [3]. These examples illustrate the physical and mental health benefits people can experience from spending time in various natural environments. The biophilia hypothesis posits that humans possess a high innate affinity for nature, which is a trait modern humans share with our ancestors [4]. By gravitating towards nature, individuals may envision a future in which they can thrive [5], optimise their abilities, and enhance their overall well-being [6].

Evidence supporting this preference for nature can also be observed in visual attention bias. In a previous study using the dot-probe task, probes replacing natural landscapes dominated by vegetation were detected more quickly than those replacing urban landscapes [7]. Schiebel et al. [8] further confirmed participants’ inclination to approach nature and avoid urban environments using the dot-probe task, implicit association test, and approach-avoidance task. Natural landscapes capture more human attention than urban landscapes, effortlessly guiding one’s focus towards nature [9].

The animate monitoring hypothesis (AMH) posits that, within the realm of nature, animate objects possess an attentional advantage [10]. The group-foraging nature of early humans meant that rapid and frequent monitoring of both humans and animals was essential because their situations could change quickly. The AMH has been validated through a range of tasks. For example, in the change-detection task [10], changes involving humans and animals were found to be more frequently and swiftly detected than changes related to vehicles, buildings, plants, or tools, and were less prone to being overlooked. Furthermore, in the visual search task [11], animals were detected more rapidly than inanimate objects, while in the attentional blink task [12], the duration of undetectability of new stimuli following initial detection was shorter for animals than for non-animals. In addition, a comparison of the detection performance between artificial intelligence (AI) and humans revealed that while AI and humans performed equally well in detecting cars, humans outperformed AI in detecting humans [13]. These findings suggest that humans excel at recognising object categories of evolutionary significance. As noted above, accumulating evidence supports the AMH.

In contrast, the concept of plant awareness disparity (PAD) [14], formerly known as plant blindness, has been gaining wide recognition. Wandersee and Schussler [15] raised concerns that the United States displayed less interest in plants than animals, thereby negatively affecting the public’s scientific literacy. They initiated a campaign to enhance the public’s understanding of plants, attributing PAD not only to a preference for animals but also to visual perception mechanisms that make plants more likely to be overlooked. In practice, participants have been found to recall more animals than plants in recall tasks [16,17], and more readily detect animals compared with plants in the attentional blink task [18]. These studies underscore that PAD is reflected not only in people’s knowledge and interests but also in their behaviour.

Considering the three theories of biophilia, AMH, and PAD, visual attention likely prioritises animals, followed by plants and then non-living organisms. Previous studies have reported findings consistent with this hypothesis. Jackson and Calvillo [19] found that in a visual search task, humans and animals, body parts and fruits, and tools and vehicles were detected the quickest, in that order. They also suggested that evolutionary relatedness, which indicates the extent to which humans are exposed to relevant stimuli throughout the evolutionary process, may significantly affect visual information processing. Although differences in attentional tendencies among the three groups were noted, a comprehensive understanding of the underlying mechanisms is still needed. One perspective indicates that people may respond to the probe faster because it serves as an alerting stimulus. Conversely, the challenge of diverting attention from an alert target may delay responses to other stimuli [20]. Thus, the AMH can be interpreted in two ways: animal-related stimuli elicit faster responses because they inherently demand attention or responses to non-animal stimuli may be delayed because attention remains preoccupied with the alert animal target.

The purpose of this study was to confirm the hierarchy in visual attention towards animals, plants, and manufactured objects. This study further sought to elucidate whether this hierarchy is driven by facilitated attention or difficulty in disengaging. To achieve these aims, we used a dot-probe task capable of distinguishing between facilitated attention and attentional disengagement difficulty. Specifically, we tested three hypotheses: (1) responses to presented stimuli are faster in the order of animals, plants, and manufactured objects, (2) attentional facilitation occurs towards animals and plants, and (3) attentional facilitation is stronger for animals than for plants. If these hypotheses are supported, it would demonstrate that responses to animals are faster owing to the attraction of visual attention, and that the attracting power is more robust for animals than for plants.

## Experiment 1

### Methods

#### Participants.

The Graduate School of Sustainable System Sciences Ethics Committee at Osaka Metropolitan University approved all procedures (2022 (1) – 36). The experimental protocol adhered to the latest version of the Declaration of Helsinki. Informed consent was obtained through the participant recruitment screen on the crowdsourcing platform. Participants indicated their understanding of the experiment’s details displayed on the screen and their willingness to participate by checking a designated box. The participants received financial compensation for their involvement, and all surveys were conducted online. The recruitment period for this experiment was from May 14 to May 23, 2023.

An a priori power analysis conducted using the R package Superpower [21] determined that a sample size of 47 was needed to obtain a power of over.80 (see S1 Appendix for details). To account for potential data exclusions, 80 participants were recruited. Among the 80 participants engaged through CrowdWorks (https://crowdworks.jp), three were excluded because of errors in saving their experimental data. Thus, data were analysed from 74 participants (27 female, 46 male, 1 non-response; *M*_age_ = 41.11 years, *SD* = 8.97, range = 22–58 years) were analysed, after excluding two who failed the instructional manipulation check and one who self-reported that their experimental data should not be included in the study.

#### Stimuli.

Object categorisation involves distinct cognitive neural processes that are contingent on an object’s conceptual level [21,22]. Birds were categorised as animals, fruits as plants [23], and vehicles and tools as manufactured objects [10,19], aligning with the superordinate categorisation of Rosch et al. [24] to equalise category hierarchies. Birds, fruit, and vehicles were designated target stimuli, whereas tools were considered neutral stimuli because the previous study reported slower reaction times towards tools than the other three categories [10,19]. The stimuli for birds were selected from the class Aves in the Linnaean taxonomy [25]. The Standard Tables of Food Composition in Japan were used to select fruits [26]. Land vehicles were chosen from class 12 (Vehicles; apparatus for locomotion by land, air, or water) defined in the Nice classification of the World Intellectual Property Organization [27]. Tools were subjectively selected by the authors, ensuring no overlap with other categories. Sixteen images, all depicting tools, were used in the practice trials. The main trials included 112 images in total: 64 tools, 16 birds, 16 fruits, and 16 vehicles. There was no overlap between the images used in the practice and main trials. All stimuli were static images obtained from Pixabay (https://pixabay.com/) and were resized to 260 x 260 px.

The effect of colour information on object recognition was stronger for colour diagnostic objects (that is, objects such as fire engines and lemons that appear in a consistent colour) than for non-colour diagnostic objects (by contrast, objects such as cars and hammers that do not consistently appear in a characteristic colour) [28,29]. The influence of colour-based information on neural activity during object processing was found to occur for fruits but not for animals or tools [23]. Moreover, because colour captures attention merely by being present on the screen, regardless of stimulus category [30–33], line drawings that excluded the effects of colour were used in the experiments.

As an index of the objective visual complexity of the stimuli, the compressed image file size was used (see Supplementary Materials S1 Table). The JPEG compressed file size correlates with subjective evaluations of visual complexity, indicating that images with larger file sizes are more complex [34–37]. An analysis of variance was conducted to examine the effect of stimulus category on visual complexity, yielding significant results (see Supplementary Materials S2 Table). Post hoc *t* tests revealed that fruits were significantly more complex than birds, whereas there were no significant differences between vehicles and fruits, or vehicles and birds. Additionally, birds, fruits, and vehicles were found to be more complex than tools. For the post hoc *t*-tests, *p*-values were adjusted using the Benjamini and Hochberg method to control the false discovery rate.

#### Procedure.

This experiment used the version of the dot-probe task version developed by Koster et al. [20], which incorporates neutral trials as a baseline for reaction time and enables differentiation between facilitated attention and attentional disengagement difficulty. The experimental programme was constructed using PsychoPy ver. 2022.1.2 [38] and hosted on Pavlovia.org (https://pavlovia.org/). The total duration of the experiment was approximately 20 minutes.

**Fig 1:**
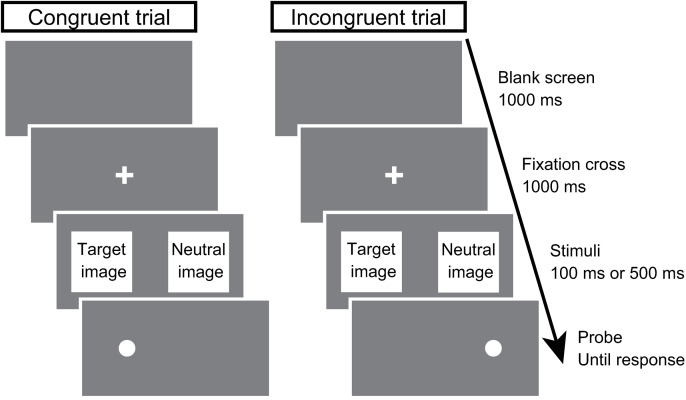
Flow of trials for the dot-probe task.

Fig 1 illustrates the sequence of a single trial. A blank screen was presented for 1000 ms, followed by a fixation point at the centre of the screen for 1000 ms. Two images were simultaneously displayed side-by-side following the disappearance of the fixation point. Stimulus onset asynchrony (SOA) was set at 100 ms and 500 ms with identical stimulus durations to assess automatic initial attention allocation and controlled attention allocation [39]. Subsequent to the image disappearing, the probe emerged in one of the two positions where the images were presented and remained visible until the participant responded. Participants were instructed to press the ‘F’ key as quickly as possible using their index finger if the probe appeared on the left side of the screen or the ‘J’ key if it appeared on the right.

The images were paired and featured either one target and one neutral stimulus or two neutral stimuli. Pairings of target and neutral stimuli were presented, and trials were considered congruent if the probe appeared in the position of the target stimulus. Trials with the probe on the opposite side were deemed incongruent. Each block comprised 56 trials arranged in a 7 (trial type: bird congruent/incongruent, fruit congruent/incongruent, vehicle congruent/incongruent, neutral) × 2 (stimulus position: left, right) × 2 (probe position: left, right) × 2 (SOA: 100, 500) design. Each image was presented once within a block and the order and pairing combinations were randomised.

Participants completed eight practice trials, followed by four blocks, totalling 224 trials. Between blocks, the participants were allowed an arbitrary rest period. An instructional manipulation check was administered after the second block to identify participants who had not earnestly engaged in the experiment [40,41]. After completing an experiment, participants were provided with the following instruction: ‘Last, it is vital to our study that we only include responses from participants who devoted their full attention to this study. Otherwise, years of effort (the researchers’ efforts and other participants’ time) could be wasted. You will receive credit for this study no matter how you answer the next item. We appreciate your honesty!’ Participants were then asked to express their preference with a simple ‘yes’ or ‘no’ response to the following question: ‘In your honest opinion, should we use your data in our analyses for this study?’ This question was designed to efficiently assess participants’ attentiveness to the experiment [42].

#### Statistical analysis.

The attentional bias index (ABI), attentional facilitation index (AFI), and disengagement index (DI) were computed based on reaction times [43,44]. The ABI was defined as the reaction time in the incongruent trials minus the reaction time in the congruent trials. A positive ABI value indicated that attention was directed towards the target stimulus (vigilance), whereas a negative value signified that attention was turned away from the target stimulus (avoidance). The AFI was calculated by subtracting the reaction time in the congruent trials from the reaction time in the neutral trials. A positive AFI indicated that attention was facilitated towards the target stimulus, whereas a negative AFI suggested inhibited processing of the target stimulus. The DI was obtained based on reaction time in the neutral trials minus the reaction time in the incongruent trials, with a negative DI indicating difficulty disengaging attention from the target stimulus [45].

A within-subjects three-factor analysis of variance (ANOVA) (factors: category, congruency, and SOA) was applied to the reaction times. For the ABI, AFI, and DI, a one-sample *t*-test was conducted with the null hypothesis set to zero. Additionally, a within-subjects two-factor ANOVA (factors: category and SOA) was used to analyse each indicator. Mendoza’s multi-sample sphericity test [46] was used before the ANOVA, and degrees of freedom were adjusted using Greenhouse–Geisser’s ε [47] for factors violating the sphericity assumption. For one-sample and post hoc *t*-tests, p-values were adjusted using the false discovery rate method proposed by Benjamini and Hochberg [48]. Analyses were conducted using R software version 4.1.3 [49] and the ‘anovakun’ function version 4.8.9 [50] for ANOVAs. The significance level was set at.05 for all analyses.

Split-half reliabilities for reaction time, ABI, AFI, and DI were obtained using the *splithalf* package [51] for R. Reliability estimates were based on 5000 random splits and corrected with the Spearman–Brown formula [52,53].

### Results

#### Data preparation.

No participant exhibited a correct response rate below 80% (*M* = 99.7%, range = 98.2–100%) [54]. The number of incorrect trials for each participant ranged from 0 to 4 (*M* = 0.65, *SD* = 0.99) and the incorrect trials were subsequently excluded from the analysis. Trials with reaction times shorter than 200 ms or longer than 2000 ms were also excluded (*M* = 0.01, *SD* = 0.12, range = 0–1) [20]. Values more than three standard deviations from the mean in each condition for each participant were identified as outliers and excluded. The number of outliers per participant varied from 0 to 5 (*M* = 1.39, *SD* = 1.26), and overall, 104 outliers were excluded from the analysis. Based on these procedures, 0.92% of the total trials were excluded from the overall dataset.

#### Reaction time.

Table 1 shows descriptive statistics for reaction times at 100 ms and 500 ms SOA. S3 Table provides the detailed results of the ANOVAs performed on reaction times. A significant main effect of SOA was observed, indicating that reaction times were slower in the 100 ms SOA condition (*M* = 479.6 ms, 95% CI [468.8, 490.4], *SD* = 115.6) compared to the 500 ms condition (*M* = 449.5 ms, 95% CI [439.3, 459.6], *SD* = 109.1; *F*(1, 73) = 165.26, *p* < .001, *η*_*p*_^2^ = .694). Furthermore, a significant main effect of category was identified with adjustments made to the interaction between category and congruency. However, the main effect of congruency and the other interaction effects were not significant. For congruent trials, reaction times were slower for vehicles (*M* = 472.3 ms, 95% CI [453.7, 490.9], *SD* = 114.5) than for birds (*M* = 461.7 ms, 95% CI [443.1, 480.4], *SD* = 115.0; *t*(147) = −6.24, *p* < .001, *d*_z_ = −.092) or frui*t*s (**M* *= 462.0 ms, 95% CI [443.9, 480.0], *SD* = 111.2; *t*(147) = 5.15, *p* < .001, *d*_z_ = .091), with no significan*t* difference between birds and fruits(*t*(147) = −0.13, *p* = .896, *d*_z_ = −.002). Reaction times for vehicles were slower in congruen*t* trials (*M* = 472.3 ms, 95% CI [453.7, 490.9], *SD* = 114.5) than in incongruent trials (*M* = 463.3 ms, 95% CI [445.1, 481.5], *SD* = 112.1; *t*(147) = 4.83, *p* < .001, *d*_z_ = .079). The simple effect of category on incongruen*t* trials and the simple effect of congruency on birds and fruits were not significant.

**Table 1:** Descriptive statistics for reaction times (ms) in Experiment 1.

Category	Congruency	100 ms SOA				500 ms SOA			
		*M*	95% CI [Low, High]		*SD*	*M*	95% CI [Low, High]		*SD*
Tool	Neutral	478.8	450.8	506.8	121.0	448.8	423.0	474.6	111.2
Bird	Congruent	478.3	450.8	505.7	118.5	445.2	419.8	470.6	109.7
	Incongruent	478.4	451.0	505.7	118.0	447.9	423.3	472.5	106.2
Fruit	Congruent	475.8	450.1	501.5	111.1	448.1	422.6	473.7	110.3
	Incongruent	478.8	451.7	505.9	116.8	450.8	424.5	477.1	113.6
Vehicle	Congruent	488.2	460.8	515.7	118.3	456.4	431.1	481.6	108.9
	Incongruent	478.1	451.7	504.6	114.0	448.4	423.2	473.7	108.9

*Note*. SOA = stimulus onset asynchrony.

The permutation-based split-half reliability for reaction time was very high across all categories, regardless of SOA or congruency (Spearman–Brown reliability = 0.98, 95% CI [0.95, 0.99], see Supplementary Materials S4 Table for details).

#### Attention bias index.

**Fig 2:**
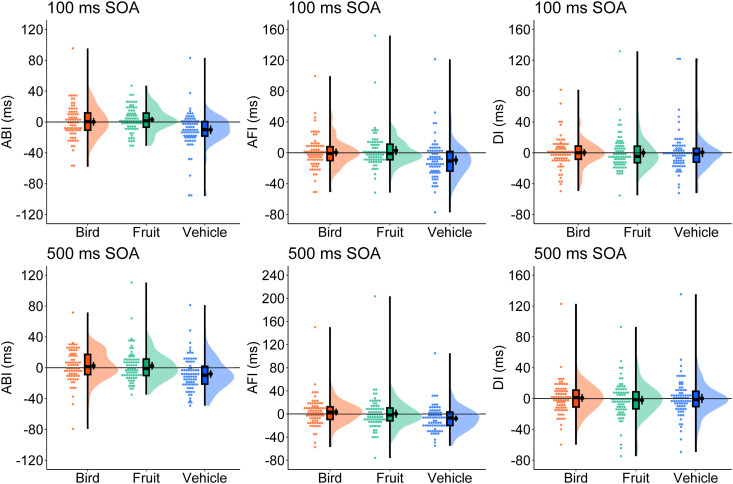
Raincloud plots of the attentional bias index (ABI, left), attentional facilitation index (AFI, centre), and disengagement index (DI, right) in Experiment 1. The results in the 100 ms SOA condition are in the top row, and the results in the 500 ms SOA condition are in the bottom row. Black dots in the cloud indicate mean values and error bars indicate 95% confidence intervals. SOA = stimulus onset asynchrony.

Fig 2 presents the results of the ABI, AFI, and DI calculations. The detailed results of the one-sample t-test performed on each indicator are shown in S5 Table, and the detailed results of the ANOVA are shown in S6 Table. The one-sample *t*-test results revealed negative ABI values for vehicles in the 100 ms and 500 ms SOA conditions. No significant ABIs were observed for either birds or fruits. An ANOVA demonstrated a significant main effect of category, indicating that the ABI was smaller for vehicles (*M* = −9.0 ms, 95% CI [−12.7, −5.3], *SD* = 22.7) than for birds (*M* = 1.4 ms, 95% CI [−2.1, 5.0], *SD* = 21.8; *t*(147) = 4.43, *p* < .001, *d*_z_ = .469) or fruits (*M* = 2.8 ms, 95% CI [−0.2, 5.9], *SD* = 18.9; *t*(147) = −4.47, *p* < .001, *d*_z_ = −.568). However, no significan*t* differences were found between birds and fruits (*t*(147) = −0.57, *p* = .569, *d*_z_ = −.069). The main effects of SOA and *t*he interaction between category and SOA were not significant. These results suggest that participants’ attention was drawn towards neutral stimuli rather than vehicles. However, it remains unclear whether this was due to attention facilitation in the neutral condition or avoidance of vehicles. This will be further investigated in the subsequent analyses of AFI and DI.

The outcomes of a one-sample *t*-test showed a negative AFI for vehicles in both the 100 ms and 500 ms SOA conditions, whereas no significant AFI was observed for birds or fruits. An ANOVA indicated a significant main effect of category; however, the main effects of SOA and the interaction between category and SOA were insignificant. Post hoc comparisons revealed that vehicle processing (*M* = −8.5 ms, 95% CI [−12.4, −4.6], *SD* = 24.1) was more inhibited than bird (*M* = 2.1 ms, 95% CI [−1.8, 5.9], *SD* = 23.8; *t*(147) = 6.24, *p* < .001, *d*_z_ = .441) or fruit processing (*M* = 1.8 ms, 95% CI [−2.8, 6.4], *SD* = 28.4; *t*(147) = −5.15, *p* < .001, *d*_z_ = −.389), wi*t*h no significant differences between birds and fruits (*t*(147) = 0.13, *p* = .896, *d*_z_ = .009).

DI was not subjected to an ANOVA because a one-sample *t*-test did not yield significant values for either condition. The Spearman–Brown reliability for ABI, AFI, and DI ranged from −0.52 to 0.23, indicating low internal consistency (see Supplementary Materials S7 Table for details).

### Discussion

In Experiment 1, the dot-probe task was used to investigate attentional bias towards birds, fruits, and vehicles. In congruent trials, reaction times were longer for vehicles than for birds or fruits. Based on the ABI and AFI values, this discrepancy arose from the inhibition of vehicle processing and attentional avoidance away from vehicles. These findings were consistent across both the 100 ms and 500 ms SOA conditions, indicating that attentional avoidance occurred in automatic and controlled attention allocation.

In contrast, no discernible attentional bias was observed for birds or fruits, rendering the present results incongruent with both the AMH and PAD. Loucks et al. [55] noted that most AMH studies primarily employed mammals as stimuli, suggesting that animals bearing more remarkable similarities to humans are preferentially processed. Given that birds are represented in the brain at greater distances from humans than mammals [56], the AMH and PAD may not have been supported because of the absence of preferential processing for birds. Consequently, Experiment 2 was conducted using the same methodology but with mammals instead of birds to further explore this phenomenon.

## Experiment 2

### Methods

The procedures and statistical analyses employed in this experiment were identical to those in Experiment 1, except that mammals were used to replace birds as stimuli. The stimuli for mammals were selected from the class Mammalia in the Linnaean taxonomy [25]. A one-way ANOVA on the objective visual complexity indicated a significant effect of category. Post hoc *t* tests showed that mammals, fruits, and vehicles were more complex than tools, while no significant differences were observed between mammals, fruits, and vehicles (see Supplementary Materials S8 and S9 Tables for details).

#### Participants.

The Graduate School of Sustainable System Sciences Ethics Committee at Osaka Metropolitan University approved all procedures (2023 (1) – 14). The experimental protocol adhered to the latest version of the Declaration of Helsinki. Informed consent was obtained through the participant recruitment screen on the crowdsourcing platform. Participants indicated their understanding of the experiment’s details displayed on the screen and their willingness to participate by checking a designated box. The participants received financial compensation for their involvement, and all surveys were conducted online. Participants were recruited between November 8 and November 21, 2023.

Eighty participants took part in the experiment via CrowdWorks. Four participants who made errors in storing the experimental data and one who failed the instructional manipulation check were excluded from the analysis. None of the participants self-reported that their experimental data should not be included in the analysis. Ultimately, data from 75 participants (38 female and 37 male; *M*_age_ = 44.39 years, *SD* = 9.45, range = 24–67 years) were included in the analysis.

### Results

#### Data preparation.

All participants achieved a correct response rate exceeding 80% (*M* = 99.7%, range = 95.5–100%). The number of incorrect responses for each participant ranged from 0 to 10 (*M* = 0.77, *SD* = 1.55). Similarly, the number of trials with reaction times faster than 200 ms or slower than 2000 ms ranged from 0 to 5 for each participant (*M* = 0.12, *SD* = 0.61). In addition, for each condition and participant, between 0 and 6 trials (*M* = 1.45, *SD* = 1.51) were more than 3 standard deviations from the mean, totalling 109 trials. Based on these procedures, 1.05% of the total dataset was excluded from analysis.

#### Reaction time.

Table 2 shows the descriptive statistics for reaction times in each condition. Detailed results of the ANOVA for the reaction times are shown in S10 Table. A significant main effect was observed for SOA, with 100 ms trials (*M* = 441.0 ms, 95% CI [435.4, 446.6], *SD* = 60.4) exhibiting slower reaction times than 500 ms trials (*M* = 412.2 ms, 95% CI [407.0, 417.4], *SD* = 56.0; *F*(1, 74) = 209.51, *p* < .001, *η*_*p*_^2^ = .739). The main effects of category and congruency were also significant but influenced by the interaction between category and congruency. No other interactions were significant. In congruent trials, vehicles (*M* = 434.2 ms, 95% CI [424.7, 443.8], *SD* = 59.3) elicited slower responses than mammals (*M* = 426.6 ms, 95% CI [416.5, 436.7], *SD* = 62.3; *t*(149) = −4.27, *p* < .001, *d*_z_ = −.124) or frui*t*s (*M* = 426.6 ms, 95% CI [416.9, 436.3], *SD* = 59.9; *t*(149) = 5.78, *p* < .001, *d*_z_ = .128), with no significan*t* differences between mammals and fruits (*t*(149) = 0.00, *p* = 1.000, *d*_z_ = .000). Response times for vehicles were slower in congruent *t*rials (*M* = 434.2 ms, 95% CI [424.7, 443.8], *SD* = 59.3) than in incongruent trials (*M* = 423.9 ms, 95% CI [414.6, 433.1], *SD* = 57.3; *t*(149) = −8.03, *p* < .001, *d*_z_ = −.177). A similar trend was observed for mammals (*M*_Incongruent_ = 423.5 ms, 95% CI [413.4, 433.6], *SD* = 62.6; *M*_Congruent_ = 426.6 ms, 95% CI [416.5, 436.7], *SD* = 62.3). The simple effect of category on incongruent trials and that of congruency on fruit trials were not significant.

**Table 2:** Descriptive statistics for reaction times (ms) in Experiment 2.

Category	Congruency	100 ms SOA				500 ms SOA			
		*M*	95% CI [Low, High]		*SD*	*M*	95% CI [Low, High]		*SD*
Tool	Neutral	438.4	426.0	450.8	53.9	413.2	398.9	427.4	61.9
Mammal	Congruent	441.1	425.5	456.8	68.0	412.1	399.9	424.2	52.6
	Incongruent	440.4	425.0	455.9	67.2	406.6	394.4	418.8	52.9
Fruit	Congruent	440.1	426.9	453.4	57.6	413.0	399.3	426.8	59.6
	Incongruent	438.4	425.4	451.5	56.8	411.2	398.1	424.3	57.1
Vehicle	Congruent	448.3	435.1	461.6	57.7	420.1	406.8	433.5	58.0
	Incongruent	437.7	425.0	450.4	55.4	410.0	397.1	422.9	56.2

*Note*. SOA = stimulus onset asynchrony.

The Spearman–Brown reliability for reaction time ranged from 0.93 to 0.96 across all categories, demonstrating high reliability regardless of SOA or congruency (Spearman–Brown reliability = 0.98, 95% CI [0.95, 0.99], see Supplementary Materials S11 Table for details).

#### Attention bias index.

**Fig 3:**
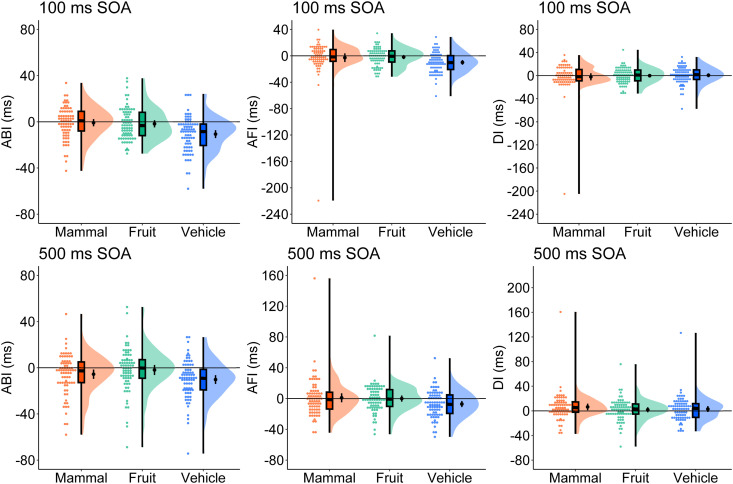
Raincloud plot of the attentional bias index (ABI), attentional facilitation index (AFI), and disengagement index (DI) in Experiment 2. The results in the 100 ms SOA condition are in the top row, and the results in the 500 ms SOA condition are in the bottom row. Black dots in the cloud indicate mean values and error bars indicate 95% confidence intervals. SOA = stimulus onset asynchrony.

Fig 3 shows the ABI, AFI, and DI distributions. Detailed results of the one-sample *t*-test and ANOVA are presented in S12 and S13 Tables. Results from one-sample *t*-tests for ABI indicated a negative bias in the vehicle 100 ms and 500 ms SOA conditions, as well as in the mammal 500 ms SOA condition. No significant ABI values were found for the other conditions. An ANOVA revealed a significant main effect of category; however, the main effect of SOA and interaction between category and SOA were not significant. Multiple comparisons showed that vehicles (*M* = −10.4 ms, 95% CI [−12.9, −7.8], *SD* = 15.8) exhibited a smaller ABI than mammals (*M* = −3.1 ms, 95% CI [−5.7, −0.5], *SD* = 15.9; *t*(149) = 3.95, *p* < .001, *d*_z_ = .459) or fruits (*M* = −1.8 ms, 95% CI [−4.4, 0.9], *SD* = 16.5; *t*(149) = −4.66, *p* < .001, *d*_z_ = −.532), wi*t*h no significant differences between mammals and fruits (*t*(149) = −0.74, *p* = .460, *d*_z_ = −.082).

One-sample *t*-tests for the AFI showed negative values only for vehicles (100 ms SOA and 500 ms). Fruits and mammals did not exhibit significant AFI values. An ANOVA indicated that the main effect of SOA and interaction between category and SOA were not significant; only the main effect of category was significant. Vehicles (*M* = −8.5 ms, 95% CI [−11.1, −5.8], *SD* = 16.4) displayed a smaller AFI compared to mammals (*M* = −0.8 ms, 95% CI [−5.2, 3.6], *SD* = 27.2; *t*(149) = 4.27, *p* < .001, *d*_z_ = .315) and fruits (*M* = −0.8 ms, 95% CI [−3.4, 1.7], *SD* = 15.7; *t*(149) = −5.78, *p* < .001, *d*_z_ = −.476), wi*t*h no significant differences between mammals and fruits (*t*(149) = 0.00, *p* < 1.000, *d*_z_ = .000).

Significantly positive one-sample *t*-test results for DI were observed only for mammals in the 500 ms SOA, with no significant values in the other trials. An ANOVA was not conducted for DI [45], as it is meaningful for negative values. The permutation-based split-half reliability for ABI, AFI, and DI ranged from −0.46 to 0.65, suggesting insufficient reliability (see Supplementary Materials S14 Table for details).

### Discussion

Overall, Experiment 2 yielded results similar to those of Experiment 1. Attention was directed away from vehicles, as indicated by the ABI, which was attributed to the inhibition of vehicle-related processing, as reflected in the AFI. Additionally, vehicles led to delayed responses in the congruent trials compared to mammals and fruits. The results for mammals and fruits mirrored those obtained for birds and fruits in Experiment 1, demonstrating the absence of significant attentional bias. The negative attentional bias identified for mammals in the 500 ms SOA contradicts the AMH. According to the AMH, humans elicit the strongest attentional advantage [10]. Therefore, we conducted a third experiment using images of humans to test whether an attentional bias would emerge.

## Experiment 3

### Methods

This experiment followed the same procedures and statistical analyses as in Experiments 1 and 2, except that human images were used as stimuli instead of birds or mammals. One-way ANOVA revealed a significant effect of category on objective visual complexity. Post-hoc comparisons indicated that humans, fruits, and vehicles were more complex than tools, with no significant differences among the former three (see Supplementary Materials, S15 and S16 Tables).

#### Participants.

All procedures were approved by the Graduate School of Sustainable System Sciences Ethics Committee of Osaka Metropolitan University (2023 (1)–14) and complied with the Declaration of Helsinki. Participants provided informed consent online via a checkbox on the crowdsourcing platform. They received monetary compensation, and all procedures were conducted online between July 23 and July 24, 2025.

Eighty individuals participated via CrowdWorks. Four were excluded: two due to data storage issues and two due to failing the instructional check. None of the participants requested data exclusion. The final sample included 76 participants (31 female and 45 male; *M*_age_ = 43.05 years, *SD* = 8.50, range = 23–63 years).

### Results

#### Data preparation.

All participants had over 80% correct responses (*M* = 99.7%, range = 96.9–100%). The errors per participant ranged from 0 to 7 (*M* = 0.63, *SD* = 1.23). Trials with reaction times below 200 ms or above 2000 ms ranged from 0 to 4 per participant (*M* = 0.20, *SD* = 0.69). Trials exceeding ±3 SDs from the mean per condition ranged from 0 to 6 (*M* = 1.87, *SD* = 1.58). Across all exclusion criteria, a total of 205 trials were removed, accounting for 1.20% of the data.

#### Reaction time.

Descriptive statistics are presented in Table 3, and detailed ANOVA results in S17 Table. A main effect of SOA was found, with responses slower at 100 ms (*M* = 448.8 ms, 95% CI [442.9, 454.7], *SD* = 64.2) than at 500 ms (*M* = 424.2 ms, 95% CI [418.2, 430.2], *SD* = 65.5; *F*(1, 75) = 94.63, *p* < .001, *η*_*p*_^2^ = .558). Significant main effects of category and congruency were also observed, though these were modulated by their interaction. In congruent trials, responses to vehicles (*M* = 444.3 ms, 95% CI [433.8, 454.9], *SD* = 65.8) were slower than to humans (*M* = 433.7 ms, 95% CI [423.0, 444.3], *SD* = 66.5; *t*(151) = −5.59, *p* < .001, *d*_z_ = −.161) and frui*t*s (*M* = 436.1 ms, 95% CI [425.3, 446.9], *SD* = 67.4; *t*(151) = 4.46, *p* < .001, *d*_z_ = .123), with no difference be*t*ween the latter two. For vehicles, reaction times were slower in congruent than in incongruent trials (*t*(151) = −5.65, *p* < .001, *d*_z_ = −.140). No significant effects were found for ca*t*egory in incongruent trials or for congruency in the human and fruit conditions. Split-half reliability for reaction time was high across conditions (rs = .95–.96; see S18 Table).

**Table 3:** Descriptive statistics for reaction times (ms) in Experiment 3.

Category	Congruency	100 ms SOA				500 ms SOA			
		*M*	95% CI [Low, High]		*SD*	*M*	95% CI [Low, High]		*SD*
Tool	Neutral	449.1	433.7	464.6	67.7	421.4	407.6	435.3	60.6
Human	Congruent	444.3	430.7	458.0	59.8	423.0	406.7	439.3	71.4
	Incongruent	446.7	431.9	461.6	65.0	420.5	406.1	434.8	62.8
Fruit	Congruent	448.7	434.3	463.1	63.0	423.5	407.6	439.5	69.7
	Incongruent	448.5	433.2	463.8	66.8	423.8	408.3	439.3	67.8
Vehicle	Congruent	457.5	442.0	473.0	67.8	431.2	417.2	445.2	61.3
	Incongruent	447.2	432.6	461.7	63.8	423.3	409.3	437.2	61.0

*Note*. SOA = stimulus onset asynchrony.

#### Attention bias index.

**Fig 4:**
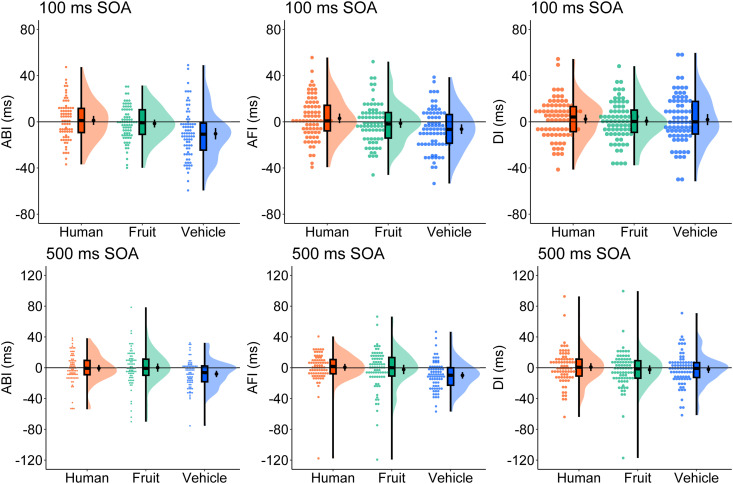
Raincloud plot of the attentional bias index (ABI), attentional facilitation index (AFI), and disengagement index (DI) in Experiment 3. The results in the 100 ms SOA condition are in the top row, and the results in the 500 ms SOA condition are in the bottom row. Black dots in the cloud indicate mean values and error bars indicate 95% confidence intervals. SOA = stimulus onset asynchrony.

Fig 4 displays the ABI, AFI, and DI distributions. One-sample t-tests (S19 Table) revealed negative ABI values for vehicles in both SOA conditions; the other conditions showed no significant biases. ANOVA results (S20 Table) showed a significant effect of category, with vehicles (*M* = −9.1 ms, 95% CI [−12.3, −5.9], *SD* = 15.8) exhibiting smaller ABI than humans (*M* = −0.1 ms, 95% CI [−3.8, 3.6], *SD* = 23.0; *t*(151) = 3.89, *p* < .001, *d*_z_ = .419) and fruits (*M* = 0.0 ms, 95% CI [−3.3, 3.4], *SD* = 20.8; *t*(151) = −3.94, *p* < .001, *d*_z_ = −.449). No difference emerged be*t*ween humans and fruits, and no main effect of SOA or interaction was found.

AFI was significantly negative only for vehicles, with no effect observed for humans or fruits. ANOVA again revealed only a category effect: vehicles (*M* = −9.0 ms, 95% CI [−12.3, −5.8], *SD* = 20.1) showed smaller AFI than both humans (*M* = 1.6 ms, 95% CI [−2.5, 5.7], *SD* = 25.8; *t*(151) = 5.59, *p* < .001, *d*_z_ = .454) and fruits (*M* = −0.9 ms, 95% CI [−4.8, 3.1], *SD* = 24.6; *t*(151) = −4.46, *p* < .001, *d*_z_ = −.361). No significan*t* difference was found between humans and fruits.

DI values were non-significant across conditions, and no ANOVA was conducted. Reliability estimates for ABI, AFI, and DI were low (rs = −0.20 to 0.47; see S21 Table).

### Discussion

Experiment 3 replicated the findings of Experiments 1 and 2, showing delayed responses and attentional avoidance of vehicles. Even when humans were used as stimuli, no attentional bias towards them was observed, mirroring the results previously found for birds and mammals.

## General discussion

The current study posited that attention would be preferentially directed towards animals, plants, and manufactured objects, in that order. Attentional bias was investigated through a dot-probe task. In Experiment 1, which included birds, fruits, vehicles, and tools as stimuli, no attentional bias towards birds and fruits was detected. Attentional avoidance and slower responses were instead evident when vehicles were involved. Experiment 2 and 3 involved mammals and humans as stimuli, which have been suggested to possess an attentional advantage over birds. However, the results were nearly identical to those of Experiment 1. Consequently, the three hypotheses of the present study were not supported, although the findings do offer valuable insights into attentional inclinations towards animate and inanimate objects.

The absence of a difference in attentional bias between flora, fauna, and tools contradicts the findings of previous studies [8,57], in which participants tended to focus more on nature than on cities. This discrepancy could be attributed to the stimuli used. In the current study, mammals and fruits were presented to participants to align the hierarchy of conceptual categories across stimuli [24]. However, natural landscapes, in contrast to urban landscapes, are characterised by their richness in colour and fractality. Fractality encompasses entire landscapes rather than individual objects [58], and several studies on biophilia have utilised landscapes containing multiple elements as experimental stimuli [59,60]. The high-level processing that elicits mental images of the natural environment is efficient for perceptual recovery [61]; therefore, stimuli with limited natural properties that extract elements from the landscape may lack the restorative elements and attractiveness necessary to capture people’s attention. Even if the stimuli consist of extracted elements, the results may differ if, for example, flowers, which are known for their psychologically restorative effects [62], were shown. Nevertheless, the present study used fruits in accordance with previous attention research. Caution should be exercised when interpreting the relationship between biophilia and attention, and future experiments that employ landscapes that align with the natural requirements associated with biophilia are warranted.

The current findings of a lack of significant differences in reaction times for animals compared to those for fruits and tools contradict the AMH. Studies employing change detection [10], visual search [11], and attentional blink tasks [12] have supported the AMH; however, investigations using flicker [63] and multiple object tracking tasks [64] have failed to demonstrate an attentional advantage towards animals. Differential attentional patterns across experimental tasks have also been observed in research on threat perception [65] and food [66]. This variability may be attributed to the multifaceted nature of assessing human visual and cognitive functions, including attention, memory, decision-making, reward processing, and spatial vision [67]. The fact that the AMH did not manifest in the dot-probe task involving humans or mammals lends credence to the notion that animal advantage does not primarily stem from attention, but may be attributable to other factors, such as the ease of short-term memory retention and encoding [68].

The present results demonstrated no discernible differences between animals and fruits, implying that PAD is not primarily rooted in variations in attentional bias towards animals and plants. This outcome aligns with the findings of one study that used the one-back task with line drawings and grayscale images [23]. What other factors besides attention could explain PAD? Colour cannot account for PAD, as evidenced by faster reaction times for fruits compared to animals and tools in a one-back task using colour images. Neural activity comparisons between colour and grayscale images revealed distinct neural responses to fruits but no differences for animals or tools [23]. Moreover, PAD was observed in a visual search task even when using monochrome stimuli [19]. These findings suggest that colour confers an advantage to fruits and that other factors play a more prominent role in shaping PAD. Regarding familiarity, mammals and birds, which are frequently featured in publications [69,70], are more familiar to most people than plants. Novel stimuli are more likely to capture attention than familiar stimuli [71,72], and familiarity favours plants in attentional allocation. Therefore, familiarity is unlikely to explain PAD. Considering that PAD was previously observed in a recognition task based on colour images [16, 17], this could plausibly be due to attitudinal, interest, encoding, or retaining factors.

In all three experiments, responses to vehicles were slower than those to animals and fruits. This finding partially aligns with prior research employing change detection [10] and visual search tasks [19], wherein vehicles were detected more slowly than animals but faster than other manufactured objects. Reaction time showed high internal consistency, and the raw reaction times [73] and initial attentional component measured in the 100 ms SOA condition [74] have demonstrated reasonable reliability. The observed delayed response to vehicles relative to other stimuli in the 100 ms SOA condition in all three experiments suggests a certain degree of reliability.

In the present study, encountering a vehicle inhibited perceptual processing and led to attentional avoidance, resulting in slower responses compared to the effects of encountering animals and plants. Daily life involves mild risks, such as road accidents, and healthy control participants have been found to often deliberately redirect their attention away from stimuli associated with risks below a certain threshold [45]. This is done to prevent heightened anxiety from trivial fears [75,76] or to pursue goals efficiently [77]. Although the present study did not directly assess perceived threat, the delayed responses to vehicles may tentatively reflect processing inhibition and attentional avoidance influenced by such perceptions. Nonetheless, concerns have been raised regarding the interpretation of the bias index computed in the dot-probe task [78], as well as the low reliability and congruency of the results [79]. In the present study, ABI, AFI, and DI also showed low internal consistency. The reason for the low internal consistency of bias indices was that taking the difference between two highly correlated measures reduces individual variability. When individual variability is small, the reliability coefficient tends to be low [80]. Additionally, the experiment itself constituted a within-subjects design, aiming to minimise individual differences to obtain robust results, which contributed to low internal consistency. While such experimental paradigms may not be reliable as measures of individual differences, they can be applicable in studies focusing on within-subject differences [81,82]. To enhance the robustness and stability of the attentional suppression and delayed response effects on vehicles, future research should validate these results by utilising event-related markers of spatial attention (e.g., N2pc components) or eye-tracking indices.

The results of the present study allow for an alternative interpretation. Of the 112 stimulus images used in the experiment, 64 were tools, and the number of tool images was greater than that for other categories. People tend to expect that the stimuli they are frequently exposed to will likely reappear [83]. Performance can improve when expected events occur [84,85], and it is possible that the expectation of tools accelerated the response to them. The reason why prioritised attention to animals and plants was not observed might be that responses to tools were quicker owing to this expectation. Even if the expectation effect on tools is removed, the finding that responses to vehicles are slower than those to animals and plants remains unchanged. However, avoidance of vehicles might not occur. This possibility should be tested through an experimental design that controls for the number of stimuli across categories.

This study has a limitation that warrants acknowledgement. The visual features of the stimuli may have introduced bias into the results. Among visual features, colour was excluded by using line drawings. Statistical tests on visual complexity showed that vehicles were not more complex than animals or plants. However, factors such as power spectrum, luminance, contrast, and shape were not controlled. These low-level visual features do not affect the superiority of animals in the visual search task [11], but it is unclear whether they have an effect in the dot-probe task. Additionally, line drawings may have excluded other important features, such as fractality, that are inherent in natural photographs. These features could play a significant role in attentional processes, and their absence might have influenced our findings. Future experiments that control for these visual features using text stimuli, image statistics [86], or photographs could clarify whether the results of this study were driven by lower-level or higher-level processes.

## Conclusion

This study aimed to elucidate the hierarchy in visual attention towards animals, plants, and manufactured objects, and to determine whether this hierarchy was due to facilitated attention or disengagement difficulty. Consistently across all three experiments, responses to vehicles were slower than those to animals and plants. Contrary to our hypotheses, no attentional biases for animals and plants were observed. Unexpectedly, vehicles inhibited perceptual processing and attention, resulting in slower responses. These findings suggest that attentional hierarchies may not be solely driven by evolutionary relevance of stimuli, and that certain man-made objects may actively suppress attention. These results may have been influenced by the imbalance in the number of stimuli, warranting further investigation.

## Supporting information

S1 Appendix R codes used for A priori power analysis. (DOCX)

S1 Table The objective visual complexity of the stimuli in Experiment 1 based on the JPEG compressed file size. (DOCX)

S2 Table Analysis of variance results for visual complexity of the stimuli in Experiment 1. (DOCX)

S3 Table Analysis of variance results for reaction times in Experiment 1. (DOCX)

S4 Table Spearman-Brown reliability for reaction times (ms) in Experiment 1. (DOCX)

S5 Table Results of one-sample t-tests on attentional tendency indices in Experiment 1. (DOCX)

S6 Table Results of analysis of variance for the attentional bias index and attentional facilitation index in Experiment 1. (DOCX)

S7 Table Spearman-Brown reliability for ABI, AFI, and DI in Experiment 1. (DOCX)

S8 Table The objective visual complexity of the stimuli in Experiment 2 based on the JPEG compressed file size. (DOCX)

S9 Table Analysis of variance results for visual complexity of the stimuli in Experiment 2. (DOCX)

S10 Table Analysis of variance results for reaction times in Experiment 2. (DOCX)

S11 Table Spearman-Brown reliability for reaction times in Experiment 2. (DOCX)

S12 Table Results of one-sample t-tests on attentional tendency indices in Experiment 2. (DOCX)

S13 Table Results of analysis of variance for the attentional bias index and attentional facilitation index in Experiment 2. (DOCX)

S14 Table Spearman-Brown reliability for ABI, AFI, and DI in Experiment 2. (DOCX)

S15 Table The objective visual complexity of the stimuli in Experiment 3 based on the JPEG compressed file size. (DOCX)

S16 Table Analysis of variance results for visual complexity of the stimuli in Experiment 3. (DOCX)

S17 Table Analysis of variance results for reaction times in Experiment 3. (DOCX)

S18 Table Spearman-Brown reliability for reaction times in Experiment 3. (DOCX)

S19 Table Results of one-sample t-tests on attentional tendency indices in Experiment 3. (DOCX)

S20 Table Results of analysis of variance for the attentional bias index and attentional facilitation index in Experiment 3. (DOCX)

S21 Table Spearman-Brown reliability for ABI, AFI, and DI in Experiment 3. (DOCX)
