# Supplementary material for: Vehicle avoidance: The hierarchy of visual attention towards animals, plants, and vehicles
Source: PLoS One. 2025 Sep 22;20(9):e0330475. doi: 10.1371/journal.pone.0330475 (PMC12453235; doi:10.1371/journal.pone.0330475)
Supplement: S1 Appendix — (DOCX) [file pone.0330475.s001.docx]

**S1 Appendix. R codes used for A priori power analysis.**

The assumed reaction times and SDs required for the sample size calculations were determined by the authors on the basis of previous studies (Albery et al., 2021; Koster et al., 2004).

**References**

Albery, I. P., Spada, M. M., & Nikčević, A. V. (2021). The COVID-19 anxiety syndrome and selective attentional bias towards COVID-19-related stimuli in UK residents during the 2020–2021 pandemic. Clinical Psychology & Psychotherapy, 28(6), 1367–1378. <https://doi.org/10.1002/cpp.2639>

Koster, E. H. W., Crombez, G., Verschuere, B., & De Houwer, J. (2004). Selective attention to threat in the dot probe paradigm: differentiating vigilance and difficulty to disengage. Behaviour Research and Therapy, 42(10), 1183-1192. https://doi.org/https://doi.org/10.1016/j.brat.2003.08.001

**## The following lines describe the R code used for the calculation.**

library(Superpower)

design_result <- ANOVA_design(design = "3w*2w*2w",

n = 47,

mu = c(410, 460, 450, 500, 430, 470, 450, 500, 450, 480, 450, 500),

sd = 65,

r <- 0.5,

plot = TRUE,

labelnames = c("Category","Bird","Fruit","Vehicle",

"Congruency","Congruent","Incongruent",

"SOA","100 ms SOA","500 ms SOA"))

ANOVA_exact(design_result,

verbose = FALSE)$main_results
