# Supplementary material for: Vehicle avoidance: The hierarchy of visual attention towards animals, plants, and vehicles
Source: PLoS One. 2025 Sep 22;20(9):e0330475. doi: 10.1371/journal.pone.0330475 (PMC12453235; doi:10.1371/journal.pone.0330475)
Supplement: S2 Table — (DOCX) [file pone.0330475.s003.docx]

| **S2 Table. Analysis of variance results for visual complexity of the stimuli in Experiment 1.** | | | | | | |
| --- | --- | --- | --- | --- | --- | --- |
| **Analysis of variance** | ***F*** | ***df*** | ***p*** | ***η_p_*^2^** |  | |
| Category | 12.47 | 3, 108 | <.001 | .257 |  | |
| **Post hoc *t* tests** | ***t*** | ***df*** | ***p*** | ***dz*** | **95% CI [Low, High]** | |
| Bird vs Fruit | -2.52 | 19.84 | .030 | -0.892 | -0.135 | -1.649 |
| Bird vs Vehicle | -0.37 | 24.74 | .713 | -0.132 | -0.854 | 0.591 |
| Bird vs Tool | 4.04 | 50.96 | <.001 | 0.759 | 0.190 | 1.328 |
| Fruit vs Vehicle | 2.02 | 26.22 | .064 | 0.714 | -0.030 | 1.459 |
| Fruit vs Tool | 4.70 | 20.73 | <.001 | 1.450 | 0.849 | 2.051 |
| Vehicle vs Tool | 3.37 | 28.34 | .004 | 0.817 | 0.246 | 1.388 |
