# Supplementary material for: Vehicle avoidance: The hierarchy of visual attention towards animals, plants, and vehicles
Source: PLoS One. 2025 Sep 22;20(9):e0330475. doi: 10.1371/journal.pone.0330475 (PMC12453235; doi:10.1371/journal.pone.0330475)
Supplement: S3 Table — (DOCX) [file pone.0330475.s004.docx]

| **S3 Table. Analysis of variance results for reaction times in Experiment 1.** | | | | | | | |
| --- | --- | --- | --- | --- | --- | --- | --- |
|  | **Sphericity test** | | | **Analysis of variance** | | | |
|  | ***χ*^2^ (2)** | ***ε*** | ***p*** | ***F*** | ***df*** | ***p*** | ***η_p_*^2^** |
| Category | 12.17 | 0.865 | .002 | 7.54 | 1.73, 126.35 | .001 | .094 |
| Congruency | - | 1.000 | - | 2.84 | 1, 73 | .096 | .037 |
| SOA | - | 1.000 | - | 165.26 | 1, 73 | < .001 | .694 |
| Category × Congruency | 5.14 | 0.936 | .076 | 10.72 | 1.87, 136.58 | < .001 | .128 |
| Category×SOA | 0.03 | 1.000 | .987 | 1.37 | 2, 146 | .259 | .018 |
| Congruency × SOA | - | 1.000 | - | 0.53 | 1, 73 | .468 | .007 |
| Category × Congruency × SOA | 0.54 | 0.993 | .765 | 0.27 | 2, 146 | .767 | .004 |
| **Simple effects** |  |  |  |  |  |  |  |
| Congruent: Category | 9.85 | 0.887 | .007 | 16.97 | 1.77, 129.45 | < .001 | .189 |
| Incongruent: Category | 6.29 | 0.923 | .043 | 0.42 | 1.85, 134.73 | .639 | .006 |
| Vehicle: Congruency | - | 1.000 | - | 19.61 | 1, 73 | < .001 | .212 |
| Bird: Congruency | - | 1.000 | - | 0.60 | 1, 73 | .441 | .008 |
| Fruit: Congruency | - | 1.000 | - | 2.74 | 1, 73 | .102 | .036 |
| **Post hoc *t* tests** | ***t* (147)** | ***p*** | ***dz*** | **95% CI [Low, High]** | |  |  |
| Congruent: Vehicle vs Bird | -6.24 | < .001 | -0.092 | -0.121 | -0.063 |  |  |
| Congruent: Vehicle vs Fruit | 5.15 | < .001 | 0.091 | 0.056 | 0.126 |  |  |
| Congruent: Bird vs Fruit | -0.13 | .896 | -0.002 | -0.033 | 0.028 |  |  |
| Vehicle: Congruent vs Incongruent | 4.83 | < .001 | 0.079 | 0.047 | 0.111 |  |  |

*Note*. SOA = stimulus onset asynchrony.
