# Supplementary material for: Vehicle avoidance: The hierarchy of visual attention towards animals, plants, and vehicles
Source: PLoS One. 2025 Sep 22;20(9):e0330475. doi: 10.1371/journal.pone.0330475 (PMC12453235; doi:10.1371/journal.pone.0330475)
Supplement: S5 Table — (DOCX) [file pone.0330475.s006.docx]

| S5 Table. Results of one-sample t-tests on attentional tendency indices in Experiment 1. | | | | | | | | | | |
| --- | --- | --- | --- | --- | --- | --- | --- | --- | --- | --- |
| ABI | **Category** | ***M*** | **95% CI [Low, High]** | | ***SD*** | **One-sample *t*-test** | | | | |
|  |  |  |  | |  | ***t* (73)** | ***p*** | ***dz*** | **95% CI [Low, High]** | |
| 100 ms SOA | Bird | 0.1 | -5.0 | 5.3 | 22.3 | 0.05 | .959 | 0.006 | -0.457 | 0.469 |
|  | Fruit | 3.0 | -0.7 | 6.7 | 15.9 | 1.62 | .217 | 0.189 | -0.276 | 0.653 |
|  | Vehicle | -10.1 | -15.6 | -4.6 | 23.8 | -3.66 | .003 | -0.425 | -0.894 | 0.043 |
| 500 ms SOA | Bird | 2.7 | -2.2 | 7.6 | 21.3 | 1.10 | .349 | 0.128 | -0.336 | 0.592 |
|  | Fruit | 2.7 | -2.3 | 7.7 | 21.7 | 1.07 | .349 | 0.124 | -0.340 | 0.588 |
|  | Vehicle | -7.9 | -13.0 | -2.9 | 21.7 | -3.14 | .007 | -0.364 | -0.832 | 0.103 |
| AFI | **Category** | ***M*** | **95% CI [Low, High]** | | ***SD*** | **One-sample *t*-test** | | | | |
|  |  |  |  | |  | ***t* (73)** | ***p*** | ***dz*** | **95% CI [Low, High]** | |
| 100 ms SOA | Bird | 0.5 | -4.5 | 5.6 | 21.9 | 0.21 | .852 | 0.024 | -0.439 | 0.488 |
|  | Fruit | 3.0 | -2.9 | 8.9 | 25.3 | 1.01 | .472 | 0.118 | -0.346 | 0.581 |
|  | Vehicle | -9.5 | -15.4 | -3.5 | 25.8 | -3.15 | .014 | -0.366 | -0.833 | 0.101 |
| 500 ms SOA | Bird | 3.6 | -2.3 | 9.5 | 25.6 | 1.21 | .463 | 0.140 | -0.324 | 0.604 |
|  | Fruit | 0.7 | -6.6 | 7.9 | 31.3 | 0.19 | .852 | 0.022 | -0.442 | 0.485 |
|  | Vehicle | -7.5 | -12.7 | -2.4 | 22.4 | -2.90 | .015 | -0.337 | -0.804 | 0.130 |
| DI | **Category** | ***M*** | **95% CI [Low, High]** | | ***SD*** | **One-sample *t*-test** | | | | |
|  |  |  |  | |  | ***t* (73)** | ***p*** | ***dz*** | **95% CI [Low, High]** | |
| 100 ms SOA | Bird | 0.4 | -4.4 | 5.2 | 20.6 | 0.17 | .996 | 0.020 | -0.444 | 0.483 |
|  | Fruit | 0.0 | -5.5 | 5.5 | 23.7 | -0.01 | .996 | -0.001 | -0.464 | 0.463 |
|  | Vehicle | 0.7 | -5.6 | 6.9 | 27.0 | 0.21 | .996 | 0.024 | -0.439 | 0.488 |
| 500 ms SOA | Bird | 0.9 | -4.3 | 6.1 | 22.3 | 0.34 | .996 | 0.039 | -0.424 | 0.503 |
|  | Fruit | -2.0 | -7.6 | 3.6 | 24.3 | -0.70 | .996 | -0.082 | -0.545 | 0.382 |
|  | Vehicle | 0.4 | -5.6 | 6.3 | 25.6 | 0.13 | .996 | 0.015 | -0.449 | 0.478 |

*Note*. ABI = attentional bias index; AFI = attentional facilitation index; DI = disengagement index; SOA = stimulus onset asynchrony.
