# Supplementary material for: Vehicle avoidance: The hierarchy of visual attention towards animals, plants, and vehicles
Source: PLoS One. 2025 Sep 22;20(9):e0330475. doi: 10.1371/journal.pone.0330475 (PMC12453235; doi:10.1371/journal.pone.0330475)
Supplement: S6 Table — (DOCX) [file pone.0330475.s007.docx]

| S6 Table. Results of analysis of variance for the attentional bias index and attentional facilitation index in Experiment 1. | | | | | | | |  |
| --- | --- | --- | --- | --- | --- | --- | --- | --- |
| ABI | **Sphericity test** | | | **Analysis of variance** | | | |  |
|  | ***χ*^2^ (2)** | ***ε*** | ***p*** | ***F*** | ***df*** | ***p*** | ***η_p_*^2^** |  |
| Category | 5.14 | 0.936 | .076 | 10.72 | 1.87, 136.58 | < .001 | .128 |  |
| SOA | - | 1.000 | - | 0.53 | 1, 73 | .468 | .007 |  |
| Category×SOA | 0.54 | 0.993 | .765 | 0.27 | 2, 146 | .767 | .004 |  |
| Post hoc *t* tests | ***t* (147)** | ***p*** | ***dz*** | **95% CI [Low, High]** | |  |  |  |
| Bird vs Fruit | -0.57 | .569 | -.069 | -0.308 | 0.169 |  |  |  |
| Bird vs Vehicle | 4.43 | < .001 | .469 | 0.250 | 0.689 |  |  |  |
| Vehicle vs Fruit | -4.47 | < .001 | -.568 | -0.838 | -0.298 |  |  |  |
| AFI | **Sphericity test** | | | **Analysis of variance** | | | | |
|  | ***χ*^2^ (2)** | ***ε*** | ***p*** | ***F*** | ***df*** | ***p*** | ***η_p_*^2^** | |
| Category | 9.85 | 0.887 | .007 | 16.97 | 1.77, 129.45 | < .001 | .189 | |
| SOA | - | 1.000 | - | 0.06 | 1, 73 | .807 | .001 | |
| Category×SOA | 1.26 | 0.983 | .533 | 1.65 | 2, 146 | .196 | .022 | |
| Post hoc *t* tests | ***t* (147)** | ***p*** | ***dz*** | **95% CI [Low, High]** | |  |  | |
| Bird vs Fruit | 0.13 | .896 | .009 | -0.123 | 0.141 |  |  | |
| Bird vs Vehicle | 6.24 | < .001 | .441 | 0.295 | 0.587 |  |  | |
| Vehicle vs Fruit | -5.15 | < .001 | -.389 | -0.543 | -0.235 |  |  | |

*Note*. ABI = attentional bias index; AFI = attentional facilitation index; SOA = stimulus onset asynchrony.
