# Supplementary material for: Vehicle avoidance: The hierarchy of visual attention towards animals, plants, and vehicles
Source: PLoS One. 2025 Sep 22;20(9):e0330475. doi: 10.1371/journal.pone.0330475 (PMC12453235; doi:10.1371/journal.pone.0330475)
Supplement: S7 Table — (DOCX) [file pone.0330475.s008.docx]

| **S7 Table. Spearman-Brown reliability for ABI, AFI, and DI in Experiment 1.** | | | | | | | |
| --- | --- | --- | --- | --- | --- | --- | --- |
| **Index** | **Category** | **100 ms SOA** | | | **500 ms SOA** | | |
|  |  | **SB** | **95% CI [Low, High]** | | **SB** | **95% CI [Low, High]** | |
| ABI | Bird | -0.16 | -0.62 | 0.40 | -0.06 | -0.54 | 0.39 |
|  | Fruit | -0.52 | -0.75 | -0.16 | -0.02 | -0.52 | 0.42 |
|  | Vehicle | -0.03 | -0.60 | 0.45 | -0.04 | -0.48 | 0.40 |
| AFI | Bird | -0.12 | -0.61 | 0.38 | 0.24 | -0.38 | 0.61 |
|  | Fruit | 0.21 | -0.32 | 0.57 | 0.53 | 0.08 | 0.75 |
|  | Vehicle | 0.07 | -0.50 | 0.52 | 0.02 | -0.51 | 0.45 |
| DI | Bird | -0.26 | -0.66 | 0.31 | 0.02 | -0.52 | 0.45 |
|  | Fruit | -0.08 | -0.55 | 0.43 | 0.14 | -0.43 | 0.53 |
|  | Vehicle | 0.23 | -0.32 | 0.59 | 0.23 | -0.32 | 0.58 |
| *Note*. SB = Spearman-Brown reliability. | | | | | | | |
