# Supplementary material for: Vehicle avoidance: The hierarchy of visual attention towards animals, plants, and vehicles
Source: PLoS One. 2025 Sep 22;20(9):e0330475. doi: 10.1371/journal.pone.0330475 (PMC12453235; doi:10.1371/journal.pone.0330475)
Supplement: S8 Table — (DOCX) [file pone.0330475.s009.docx]

| **S8 Table. The objective visual complexity of the stimuli in Experiment 2 based on the JPEG compressed file size.** | | | | | | | | |
| --- | --- | --- | --- | --- | --- | --- | --- | --- |
| **Category** | ***M*** | **95% CI [Low, High]** | | ***SD*** |  |  | |  |
| Tool | 8656.22 | 7674.16 | 9638.28 | 3931.50 |  |  |  |  |
| Mammal | 13493.44 | 12013.58 | 14973.29 | 2777.18 |  |  |  |  |
| Fruit | 14565.56 | 12097.34 | 17033.78 | 4632.01 |  |  |  |  |
| Vehicle | 11749.44 | 10094.44 | 13404.43 | 3105.86 |  |  |  |  |
|  | | | | | | | | |
