# Supplementary material for: Vehicle avoidance: The hierarchy of visual attention towards animals, plants, and vehicles
Source: PLoS One. 2025 Sep 22;20(9):e0330475. doi: 10.1371/journal.pone.0330475 (PMC12453235; doi:10.1371/journal.pone.0330475)
Supplement: S9 Table — (DOCX) [file pone.0330475.s010.docx]

| **S9 Table. Analysis of variance results for visual complexity of the stimuli in Experiment 2.** | | | | | | |
| --- | --- | --- | --- | --- | --- | --- |
| **Analysis of variance** | ***F*** | ***df*** | ***p*** | ***η_p_*^2^** |  | |
| Category | 15.00 | 3, 108 | <.001 | .294 |  | |
| **Post hoc *t* tests** | ***t*** | ***df*** | ***p*** | ***dz*** | **95% CI [Low, High]** | |
| Mammal vs Fruit | -0.79 | 24.55 | .435 | -0.281 | -1.006 | 0.445 |
| Mammal vs Vehicle | 1.67 | 29.63 | .126 | 0.592 | -0.146 | 1.330 |
| Mammal vs Tool | 5.69 | 31.89 | <.001 | 1.294 | 0.702 | 1.887 |
| Fruit vs Vehicle | 2.02 | 26.22 | .081 | 0.714 | -0.031 | 1.459 |
| Fruit vs Tool | 4.70 | 20.73 | <.001 | 1.450 | 0.849 | 2.051 |
| Vehicle vs Tool | 3.37 | 28.34 | .004 | 0.817 | 0.246 | 1.388 |
