# Supplementary material for: Vehicle avoidance: The hierarchy of visual attention towards animals, plants, and vehicles
Source: PLoS One. 2025 Sep 22;20(9):e0330475. doi: 10.1371/journal.pone.0330475 (PMC12453235; doi:10.1371/journal.pone.0330475)
Supplement: S10 Table — (DOCX) [file pone.0330475.s011.docx]

| **S10 Table. Analysis of variance results for reaction times in Experiment 2.** | | | | | | | |
| --- | --- | --- | --- | --- | --- | --- | --- |
|  | **Sphericity test** | | | **Analysis of variance** | | | |
|  | ***χ*^2^ (2)** | ***ε*** | ***p*** | ***F*** | ***df*** | ***p*** | ***η_p_*^2^** |
| Category | 5.15 | 0.936 | .076 | 9.24 | 1.87, 138.57 | < .001 | .111 |
| Congruency | - | 1.000 | - | 39.40 | 1, 74 | < .001 | .347 |
| SOA | - | 1.000 | - | 209.51 | 1, 74 | < .001 | .739 |
| Category×Congruency | 1.73 | 1.003 | .422 | 12.62 | 2, 148 | < .001 | .146 |
| Category×SOA | 42.54 | 0.703 | < .001 | 1.01 | 1.39, 102.66 | .343 | .014 |
| Congruency×SOA | - | 1.000 | - | 0.95 | 1, 74 | .332 | .013 |
| Category×Congruency×SOA | 1.46 | 1.000 | .482 | 1.26 | 2, 148 | .288 | .017 |
| **Simple effects** |  |  |  |  |  |  |  |
| Congruent: Category | 3.12 | 0.960 | .210 | 20.74 | 2, 148 | < .001 | .219 |
| Incongruent: Category | 0.37 | .995 | .830 | 0.52 | 2, 148 | .598 | .007 |
| Vehicle: Congruency | 5.15 | 1.000 | - | 71.58 | 1, 74 | < .001 | .492 |
| Mammal: Congruency | - | 1.000 | - | 6.22 | 1, 74 | .015 | .078 |
| Fruit: Congruency | - | 1.000 | - | 1.37 | 1, 74 | .246 | .018 |
| **Post hoc *t* tests** | ***t* (149)** | ***p*** | ***dz*** | **95% CI [Low, High]** | |  |  |
| Congruent: Vehicle vs Mammal | -4.27 | < .001 | -0.124 | -0.182 | -0.067 |  |  |
| Congruent: Vehicle vs Fruit | 5.78 | < .001 | 0.128 | 0.084 | 0.172 |  |  |
| Congruent: Mammal vs Fruit | 0.00 | 1.000 | 0.000 | -0.062 | 0.062 |  |  |
| Vehicle: Congruent vs Incongruent | -8.03 | < .001 | -0.177 | -0.220 | -0.133 |  |  |

*Note*. SOA = stimulus onset asynchrony.
