# Supplementary material for: Vehicle avoidance: The hierarchy of visual attention towards animals, plants, and vehicles
Source: PLoS One. 2025 Sep 22;20(9):e0330475. doi: 10.1371/journal.pone.0330475 (PMC12453235; doi:10.1371/journal.pone.0330475)
Supplement: S12 Table — (DOCX) [file pone.0330475.s013.docx]

| **S12 Table. Results of one-sample t-tests on attentional tendency indices in Experiment 2.** | | | | | | | | | | |  |
| --- | --- | --- | --- | --- | --- | --- | --- | --- | --- | --- | --- |
| **ABI** | **Category** | ***M*** | **95% CI [Low, High]** | | ***SD*** | **One-sample *t*-test** | | | | | |
|  |  |  |  | |  | ***t* (74)** | ***p*** | ***dz*** | **95% CI [Low, High]** | | |
| 100 ms SOA | Mammal | -0.7 | -3.9 | 2.4 | 13.7 | -0.46 | .646 | -0.053 | -0.513 | 0.407 | |
|  | Fruit | -1.7 | -4.9 | 1.4 | 13.7 | -1.09 | .422 | -0.125 | -0.586 | 0.335 | |
|  | Vehicle | -10.6 | -14.1 | -7.2 | 15.0 | -6.13 | < .001 | -0.707 | -1.182 | -0.233 | |
| 500 ms SOA | Mammal | -5.5 | -9.5 | -1.4 | 17.6 | -2.70 | .017 | -0.312 | -0.775 | 0.151 | |
|  | Fruit | -1.8 | -6.2 | 2.5 | 18.9 | -0.84 | .482 | -0.097 | -0.558 | 0.363 | |
|  | Vehicle | -10.1 | -14.0 | -6.3 | 16.7 | -5.26 | < .001 | -0.607 | -1.077 | -0.136 | |
| **AFI** | **Category** | ***M*** | **95% CI [Low, High]** | | ***SD*** | **One-sample *t*-test** | | | | | |
|  |  |  |  | |  | ***t* (74)** | ***p*** | ***dz*** | **95% CI [Low, High]** | | |
| 100 ms SOA | Mammal | -2.8 | -9.4 | 3.9 | 28.7 | -0.83 | .612 | -0.096 | -0.556 | 0.364 | |
|  | Fruit | -1.8 | -4.9 | 1.4 | 13.7 | -1.11 | .538 | -0.129 | -0.589 | 0.332 | |
|  | Vehicle | -10.0 | -13.5 | -6.4 | 15.3 | -5.62 | < .001 | -0.649 | -1.121 | -0.177 | |
| 500 ms SOA | Mammal | 1.1 | -4.8 | 7.0 | 25.6 | 0.37 | .853 | 0.043 | -0.417 | 0.503 | |
|  | Fruit | 0.1 | -3.9 | 4.2 | 17.6 | 0.05 | .959 | 0.006 | -0.454 | 0.466 | |
|  | Vehicle | -7.0 | -11.0 | -3.0 | 17.3 | -3.50 | .002 | -0.404 | -0.869 | 0.061 | |
| **DI** | **Category** | ***M*** | **95% CI [Low, High]** | | ***SD*** | **One-sample *t*-test** | | | | | |
|  |  |  |  | |  | ***t* (74)** | ***p*** | ***dz*** | **95% CI [Low, High]** | | |
| 100 ms SOA | Mammal | -2.0 | -8.3 | 4.2 | 27.1 | -0.65 | .779 | -0.075 | -0.535 | 0.385 | |
|  | Fruit | 0.0 | -3.1 | 3.1 | 13.5 | -0.03 | .977 | -0.003 | -0.464 | 0.457 | |
|  | Vehicle | 0.7 | -2.7 | 4.0 | 14.6 | 0.40 | .828 | 0.046 | -0.414 | 0.506 | |
| 500 ms SOA | Mammal | 6.6 | 1.2 | 11.9 | 23.3 | 2.45 | .101 | 0.282 | -0.180 | 0.745 | |
|  | Fruit | 1.9 | -2.1 | 6.0 | 17.7 | 0.95 | .688 | 0.110 | -0.351 | 0.571 | |
|  | Vehicle | 3.1 | -1.6 | 7.9 | 20.8 | 1.31 | .581 | 0.151 | -0.309 | 0.612 | |

*Note*. ABI = attentional bias index; AFI = attentional facilitation index; DI = disengagement index; SOA = stimulus onset asynchrony.
