# Supplementary material for: Vehicle avoidance: The hierarchy of visual attention towards animals, plants, and vehicles
Source: PLoS One. 2025 Sep 22;20(9):e0330475. doi: 10.1371/journal.pone.0330475 (PMC12453235; doi:10.1371/journal.pone.0330475)
Supplement: S13 Table — (DOCX) [file pone.0330475.s014.docx]

| **S13 Table. Results of analysis of variance for the attentional bias index and attentional facilitation index in Experiment 2.** | | | | | | | |
| --- | --- | --- | --- | --- | --- | --- | --- |
| **ABI** | **Sphericity test** | | | **Analysis of variance** | | | |
|  | ***χ*^2^ (2)** | ***ε*** | ***p*** | ***F*** | ***df*** | ***p*** | ***η_p_*^2^** |
| Category | 1.73 | 0.977 | .422 | 12.62 | 2, 148 | < .001 | .146 |
| SOA | - | 1.000 | - | 0.95 | 1, 74 | .332 | .013 |
| Category×SOA | 1.46 | 0.981 | .482 | 1.26 | 2, 148 | .288 | .017 |
| **Post hoc *t* tests** | ***t* (149)** | ***p*** | ***dz*** | **95% CI [Low, High]** | |  |  |
| Mammal vs Fruit | -0.74 | .460 | -.082 | -0.300 | 0.136 |  |  |
| Mammal vs Vehicle | 3.95 | < .001 | .459 | 0.219 | 0.699 |  |  |
| Vehicle vs Fruit | -4.66 | < .001 | -.532 | -0.773 | -0.292 |  |  |
| **AFI** | **Sphericity test** | | | **Analysis of variance** | | | |
|  | ***χ*^2^ (2)** | ***ε*** | ***p*** | ***F*** | ***df*** | ***p*** | ***η_p_*^2^** |
| Category | 3.12 | 0.960 | .210 | 20.74 | 2, 148 | < .001 | .219 |
| SOA | - | 1.000 | - | 0.78 | 1, 74 | .379 | .011 |
| Category×SOA | 24.99 | 0.775 | < .001 | 0.13 | 1.55, 114.74 | .830 | .002 |
| **Post hoc *t* tests** | ***t* (149)** | ***p*** | ***dz*** | **95% CI [Low, High]** | |  |  |
| Mammal vs Fruit | 0 | 1.000 | .000 | -0.160 | 0.160 |  |  |
| Mammal vs Vehicle | 4.27 | < .001 | .315 | 0.166 | 0.464 |  |  |
| Vehicle vs Fruit | -5.78 | < .001 | -.476 | -0.647 | -0.305 |  |  |

*Note*. ABI = attentional bias index; AFI = attentional facilitation index; SOA = stimulus onset asynchrony.
