# Supplementary material for: Vehicle avoidance: The hierarchy of visual attention towards animals, plants, and vehicles
Source: PLoS One. 2025 Sep 22;20(9):e0330475. doi: 10.1371/journal.pone.0330475 (PMC12453235; doi:10.1371/journal.pone.0330475)
Supplement: S14 Table — (DOCX) [file pone.0330475.s015.docx]

| **S14 Table. Spearman-Brown reliability for ABI, AFI, and DI in Experiment 2.** | | | | | | | |
| --- | --- | --- | --- | --- | --- | --- | --- |
| **Index** | **Category** | **100 ms SOA** | | | **500 ms SOA** | | |
|  |  | **SB** | **95% CI [Low, High]** | | **SB** | **95% CI [Low, High]** | |
| ABI | Mammal | -0.46 | -0.81 | -0.05 | 0.05 | -0.35 | 0.38 |
|  | Fruit | -0.24 | -0.51 | 0.10 | -0.13 | -0.65 | 0.36 |
|  | Vehicle | -0.15 | -0.46 | 0.22 | -0.06 | -0.48 | 0.31 |
| AFI | Mammal | 0.65 | 0.36 | 0.79 | 0.50 | -0.03 | 0.71 |
|  | Fruit | -0.22 | -0.48 | 0.14 | -0.13 | -0.66 | 0.32 |
|  | Vehicle | -0.10 | -0.40 | 0.24 | -0.05 | -0.62 | 0.38 |
| DI | Mammal | 0.57 | 0.20 | 0.74 | 0.44 | -0.19 | 0.68 |
|  | Fruit | -0.24 | -0.48 | 0.09 | -0.15 | -0.75 | 0.35 |
|  | Vehicle | -0.11 | -0.41 | 0.25 | 0.28 | -0.35 | 0.58 |
| *Note*. SB = Spearman-Brown reliability. | | | | | | | |
