# Supplementary material for: Vehicle avoidance: The hierarchy of visual attention towards animals, plants, and vehicles
Source: PLoS One. 2025 Sep 22;20(9):e0330475. doi: 10.1371/journal.pone.0330475 (PMC12453235; doi:10.1371/journal.pone.0330475)
Supplement: S16 Table — (DOCX) [file pone.0330475.s017.docx]

| **S16 Table. Analysis of variance results for visual complexity of the stimuli in Experiment 3.** | | | | | | |
| --- | --- | --- | --- | --- | --- | --- |
| **Analysis of variance** | ***F*** | ***df*** | ***p*** | ***η_p_*^2^** |  | |
| Category | 11.63 | 3, 108 | <.001 | .244 |  | |
| **Post hoc *t* tests** | ***t*** | ***df*** | ***P*** | ***dz*** | **95% CI [Low, High]** | |
| Human vs Fruit | -1.92 | 28.43 | .077 | -0.680 | -1.423 | 0.062 |
| Human vs Vehicle | -0.02 | 29.26 | .987 | -0.006 | -0.728 | 0.716 |
| Human vs Tool | 2.97 | 24.50 | .013 | 0.792 | 0.222 | 1.363 |
| Fruit vs Vehicle | 2.02 | 26.22 | .077 | 0.714 | -0.030 | 1.459 |
| Fruit vs Tool | 4.70 | 20.73 | <.001 | 1.450 | 0.849 | 2.051 |
| Vehicle vs Tool | 3.37 | 28.34 | .007 | 0.817 | 0.246 | 1.388 |
