# Supplementary material for: Vehicle avoidance: The hierarchy of visual attention towards animals, plants, and vehicles
Source: PLoS One. 2025 Sep 22;20(9):e0330475. doi: 10.1371/journal.pone.0330475 (PMC12453235; doi:10.1371/journal.pone.0330475)
Supplement: S17 Table — (DOCX) [file pone.0330475.s018.docx]

| **S17 Table. Analysis of variance results for reaction times in Experiment 3.** | | | | | | | |
| --- | --- | --- | --- | --- | --- | --- | --- |
|  | **Sphericity test** | | | **Analysis of variance** | | | |
|  | ***χ*^2^ (2)** | ***ε*** | ***p*** | ***F*** | ***df*** | ***p*** | ***η_p_*^2^** |
| Category | 8.70 | 0.900 | .013 | 14.42 | 1.8, 135.02 | < .001 | .161 |
| Congruency | - | 1.000 | - | 7.06 | 1, 75 | .010 | .086 |
| SOA | - | 1.000 | - | 94.63 | 1, 75 | < .001 | .558 |
| Category × Congruency | 6.30 | 0.925 | .043 | 14.45 | 1.85, 138.68 | < .001 | .162 |
| Category × SOA | 2.76 | 0.965 | .252 | 0.14 | 2, 150 | .865 | .002 |
| Congruency × SOA | - | 1.000 | - | 0.08 | 1, 75 | .781 | .001 |
| Category × Congruency × SOA | 2.03 | 0.974 | .362 | 1.22 | 2, 150 | .299 | .016 |
| **Simple effects** |  |  |  |  |  |  |  |
| Congruent: Category | 4.47 | 0.945 | .107 | 27.64 | 2, 150 | < .001 | .269 |
| Incongruent: Category | 8.67 | 0.901 | .013 | 1.48 | 1.8, 135.07 | .233 | .019 |
| Vehicle: Congruency | - | 1.000 | - | 28.19 | 1, 75 | < .001 | .273 |
| Human: Congruency | - | 1.000 | - | 0.00 | 1, 75 | .962 | .000 |
| Fruit: Congruency | - | 1.000 | - | 0.00 | 1, 75 | .984 | .000 |
| **Post hoc *t* tests** | ***t* (151)** | ***p*** | ***dz*** | **95% CI [Low, High]** | |  |  |
| Congruent: Vehicle vs Human | -5.59 | < .001 | -0.161 | -0.218 | -0.104 |  |  |
| Congruent: Vehicle vs Fruit | 4.46 | < .001 | 0.123 | 0.068 | 0.177 |  |  |
| Congruent: Human vs Fruit | -1.46 | .147 | -0.037 | -0.086 | 0.013 |  |  |
| Vehicle: Congruent vs Incongruent | -5.65 | < .001 | -0.140 | -0.189 | -0.091 |  |  |

*Note*. SOA = stimulus onset asynchrony.
