# Supplementary material for: Vehicle avoidance: The hierarchy of visual attention towards animals, plants, and vehicles
Source: PLoS One. 2025 Sep 22;20(9):e0330475. doi: 10.1371/journal.pone.0330475 (PMC12453235; doi:10.1371/journal.pone.0330475)
Supplement: S18 Table — (DOCX) [file pone.0330475.s019.docx]

| **S18 Table. Spearman-Brown reliability for reaction times (ms) in Experiment 3.** | | | | | | | |
| --- | --- | --- | --- | --- | --- | --- | --- |
| **Category** | **Congruency** | **100 ms SOA** | | | **500 ms SOA** | | |
|  |  | **SB** | **95% CI [Low, High]** | | **SB** | **95% CI [Low, High]** | |
| Tool | Neutral | 0.96 | 0.94 | 0.98 | 0.95 | 0.92 | 0.97 |
| Human | Congruent | 0.95 | 0.92 | 0.97 | 0.95 | 0.89 | 0.98 |
|  | Incongruent | 0.96 | 0.93 | 0.98 | 0.96 | 0.95 | 0.98 |
| Fruit | Congruent | 0.96 | 0.94 | 0.98 | 0.96 | 0.94 | 0.98 |
|  | Incongruent | 0.96 | 0.94 | 0.97 | 0.96 | 0.93 | 0.98 |
| Vehicle | Congruent | 0.95 | 0.92 | 0.97 | 0.96 | 0.94 | 0.97 |
|  | Incongruent | 0.95 | 0.93 | 0.97 | 0.95 | 0.93 | 0.97 |
| *Note*. SB = Spearman-Brown reliability. | | | | | | | |
