# Supplementary material for: Vehicle avoidance: The hierarchy of visual attention towards animals, plants, and vehicles
Source: PLoS One. 2025 Sep 22;20(9):e0330475. doi: 10.1371/journal.pone.0330475 (PMC12453235; doi:10.1371/journal.pone.0330475)
Supplement: S19 Table — (DOCX) [file pone.0330475.s020.docx]

| S19 Table. Results of one-sample t-tests on attentional tendency indices in Experiment 3. | | | | | | | | | | |
| --- | --- | --- | --- | --- | --- | --- | --- | --- | --- | --- |
| ABI | **Category** | ***M*** | **95% CI [Low, High]** | | ***SD*** | **One-sample *t*-test** | | | | |
|  |  |  |  | |  | ***t* (75)** | ***p*** | ***dz*** | **95% CI [Low, High]** | |
| 100 ms SOA | Human | 2.4 | -2.1 | 6.9 | 19.6 | 1.06 | .584 | 0.122 | -0.336 | 0.579 |
|  | Fruit | -0.2 | -4.4 | 3.9 | 18.2 | -0.11 | .911 | -0.013 | -0.470 | 0.444 |
|  | Vehicle | -10.3 | -15.2 | -5.4 | 21.5 | -4.18 | < .001 | -0.480 | -0.943 | -0.016 |
| 500 ms SOA | Human | -2.5 | -8.5 | 3.4 | 25.9 | -0.85 | .596 | -0.098 | -0.555 | 0.360 |
|  | Fruit | 0.3 | -5.0 | 5.6 | 23.3 | 0.11 | .911 | 0.013 | -0.444 | 0.470 |
|  | Vehicle | -7.9 | -12.0 | -3.8 | 18.1 | -3.80 | < .001 | -0.435 | -0.898 | 0.027 |
| AFI | **Category** | ***M*** | **95% CI [Low, High]** | | ***SD*** | **One-sample *t*-test** | | | | |
|  |  |  |  | |  | ***t* (75)** | ***p*** | ***dz*** | **95% CI [Low, High]** | |
| 100 ms SOA | Human | 4.8 | -0.6 | 10.2 | 23.7 | 1.77 | .163 | 0.202 | -0.256 | 0.661 |
|  | Fruit | 0.4 | -4.7 | 5.5 | 22.3 | 0.16 | .876 | 0.018 | -0.439 | 0.475 |
|  | Vehicle | -8.3 | -13.3 | -3.4 | 21.7 | -3.34 | .004 | -0.384 | -0.845 | 0.078 |
| 500 ms SOA | Human | -1.6 | -7.8 | 4.7 | 27.5 | -0.50 | .744 | -0.057 | -0.514 | 0.400 |
|  | Fruit | -2.1 | -8.2 | 4.0 | 26.7 | -0.69 | .741 | -0.079 | -0.536 | 0.379 |
|  | Vehicle | -9.7 | -14.0 | -5.5 | 18.5 | -4.60 | <.001 | -0.527 | -0.992 | -0.063 |
| DI | **Category** | ***M*** | **95% CI [Low, High]** | | ***SD*** | **One-sample *t*-test** | | | | |
|  |  |  |  | |  | ***t* (75)** | ***p*** | ***dz*** | **95% CI [Low, High]** | |
| 100 ms SOA | Human | 2.4 | -1.5 | 6.3 | 17.1 | 1.23 | .671 | 0.141 | -0.316 | 0.599 |
|  | Fruit | 0.6 | -3.2 | 4.5 | 17.0 | 0.32 | .746 | 0.037 | -0.420 | 0.494 |
|  | Vehicle | 2.0 | -2.9 | 6.8 | 21.1 | 0.81 | .671 | 0.093 | -0.364 | 0.550 |
| 500 ms SOA | Human | 1.0 | -4.2 | 6.1 | 22.5 | 0.37 | .746 | 0.043 | -0.414 | 0.500 |
|  | Fruit | -2.4 | -8.0 | 3.2 | 24.7 | -0.85 | .671 | -0.097 | -0.555 | 0.360 |
|  | Vehicle | -1.8 | -6.6 | 3.0 | 21.0 | -0.76 | .671 | -0.088 | -0.545 | 0.370 |

*Note*. ABI = attentional bias index; AFI = attentional facilitation index; DI = disengagement index; SOA = stimulus onset asynchrony.
