# Supplementary material for: Vehicle avoidance: The hierarchy of visual attention towards animals, plants, and vehicles
Source: PLoS One. 2025 Sep 22;20(9):e0330475. doi: 10.1371/journal.pone.0330475 (PMC12453235; doi:10.1371/journal.pone.0330475)
Supplement: S20 Table — (DOCX) [file pone.0330475.s021.docx]

| S20 Table. Results of analysis of variance for the attentional bias index and attentional facilitation index in Experiment 3. | | | | | | | |  |
| --- | --- | --- | --- | --- | --- | --- | --- | --- |
| ABI | **Sphericity test** | | | **Analysis of variance** | | | |  |
|  | ***χ*^2^ (2)** | ***ε*** | ***p*** | ***F*** | ***df*** | ***p*** | ***η_p_*^2^** |  |
| Category | 6.30 | 0.925 | .043 | 14.45 | 1.85, 138.68 | < .001 | .162 |  |
| SOA | - | 1.000 | - | 0.08 | 1, 75 | .781 | .001 |  |
| Category×SOA | 2.03 | 0.974 | .362 | 1.22 | 2, 150 | .299 | .016 |  |
| Post hoc *t* tests | ***t* (151)** | ***p*** | ***dz*** | **95% CI [Low, High]** | |  |  |  |
| Human vs Fruit | -0.05 | .958 | -.005 | -0.182 | 0.173 |  |  |  |
| Human vs Vehicle | 3.89 | < .001 | .419 | 0.198 | 0.640 |  |  |  |
| Vehicle vs Fruit | -3.94 | < .001 | -.449 | -0.684 | -0.214 |  |  |  |
| AFI | **Sphericity test** | | | **Analysis of variance** | | | | |
|  | ***χ*^2^ (2)** | ***ε*** | ***p*** | ***F*** | ***df*** | ***p*** | ***η_p_*^2^** | |
| Category | 4.47 | 0.945 | .107 | 27.64 | 2, 150 | < .001 | .269 | |
| SOA | - | 1.000 | - | 1.05 | 1, 75 | .309 | .014 | |
| Category×SOA | 4.25 | 0.947 | .120 | 0.79 | 2, 150 | .457 | .010 | |
| Post hoc *t* tests | ***t* (151)** | ***p*** | ***dz*** | **95% CI [Low, High]** | |  |  | |
| Human vs Fruit | 1.46 | .147 | .098 | -0.035 | 0.230 |  |  | |
| Human vs Vehicle | 5.59 | < .001 | .454 | 0.286 | 0.622 |  |  | |
| Vehicle vs Fruit | -4.46 | < .001 | -.361 | -0.525 | -0.197 |  |  | |

*Note*. ABI = attentional bias index; AFI = attentional facilitation index; SOA = stimulus onset asynchrony.
