# Supplementary material for: Vehicle avoidance: The hierarchy of visual attention towards animals, plants, and vehicles
Source: PLoS One. 2025 Sep 22;20(9):e0330475. doi: 10.1371/journal.pone.0330475 (PMC12453235; doi:10.1371/journal.pone.0330475)
Supplement: S21 Table — (DOCX) [file pone.0330475.s022.docx]

| **S21 Table. Spearman-Brown reliability for ABI, AFI, and DI in Experiment 3.** | | | | | | | |
| --- | --- | --- | --- | --- | --- | --- | --- |
| **Index** | **Category** | **100 ms SOA** | | | **500 ms SOA** | | |
|  |  | **SB** | **95% CI [Low, High]** | | **SB** | **95% CI [Low, High]** | |
| ABI | Human | 0.06 | -0.36 | 0.42 | 0.38 | -0.21 | 0.68 |
|  | Fruit | -0.04 | -0.45 | 0.35 | 0.27 | -0.21 | 0.59 |
|  | Vehicle | 0.08 | -0.40 | 0.43 | -0.02 | -0.35 | 0.32 |
| AFI | Human | 0.35 | -0.08 | 0.61 | 0.40 | -0.18 | 0.69 |
|  | Fruit | 0.30 | -0.15 | 0.58 | 0.47 | 0.17 | 0.68 |
|  | Vehicle | 0.14 | -0.40 | 0.48 | -0.01 | -0.37 | 0.36 |
| DI | Human | -0.17 | -0.57 | 0.25 | 0.33 | -0.03 | 0.57 |
|  | Fruit | -0.20 | -0.56 | 0.21 | 0.36 | -0.13 | 0.63 |
|  | Vehicle | 0.14 | -0.32 | 0.47 | 0.17 | -0.21 | 0.48 |
| *Note*. SB = Spearman-Brown reliability. | | | | | | | |
